# Plasma Photocatalysis: A Novel Approach for Enhanced Air Disinfection in Centralised Ventilation Systems

**DOI:** 10.3390/ma18081870

**Published:** 2025-04-19

**Authors:** Hanna Koshlak, Leonid Lobanov, Borys Basok, Tetyana Hrabova, Pavlo Goncharov

**Affiliations:** 1 Department of Sanitary Engineering, Kielce University of Technology, Aleja Tysiąclecia Państwa Polskiego, 7, 25-314 Kielce, Poland; 2 E. O. Paton Electric Welding Institute NAS of Ukraine 11, K. Malevicha Str., 03150 Kyiv, Ukraine; lobanov@nas.gov.ua (L.L.); goncharov.pavlo@gmail.com (P.G.); 3 Institute of Engineering Thermophysics of National Academy of Sciences of Ukraine, Marii Kapnist, 2a, 03057 Kyiv, Ukraine; borys.basok@gmail.com (B.B.); gtln@ukr.net (T.H.)

**Keywords:** plasma photocatalysis, air disinfection, HVAC systems, indoor air quality, TiO_2_ photocatalysis, adsorbent-catalytic granule, energy efficiency, air purification, heat recovery

## Abstract

The COVID-19 pandemic highlighted the urgent need for sustainable and scalable air disinfection technologies in HVAC systems, addressing the limitations of energy-intensive and chemically intensive conventional methods. This study developed and evaluated a pilot experimental installation integrating plasma chemistry and photocatalysis for airborne pathogen and pollutant mitigation. The installation, designed with a modular architecture to simulate real-world HVAC dynamics, employed a bipolar plasma ioniser, a TiO_2_ photocatalytic module, and an adsorption-catalytic module for ozone abatement. Characterization techniques, including SEM and BET analysis, were used to evaluate the morphology and surface properties of the catalytic materials. Field tests in a production room demonstrated a 60% reduction in airborne microflora in three days, along with effective decomposition of ozone. The research also determined the optimal electrode geometry and interelectrode distance for stable corona discharge, which is essential for efficient plasma generation. Energy-efficient design considerations, which incorporate heat recovery and heat pump integration, achieved a 7–8-fold reduction in air heating energy consumption. These results demonstrate the potential of integrated plasma photocatalysis as a sustainable and scalable approach to enhance indoor air quality in centralised HVAC systems, contributing to both public health and energy efficiency.

## 1. Introduction

The COVID-19 pandemic starkly highlighted the critical need for innovative and scalable disinfection technologies to mitigate the transmission of infectious diseases through contaminated surfaces and air. Current gold standard disinfection methods often rely on energy-intensive and chemically intensive processes, raising concerns about environmental sustainability and potential long-term health impacts. Advanced oxidation processes (AOPs), particularly photocatalysis, have emerged as a promising alternative for disinfection. Photocatalysis uses reactive oxygen species (ROS) to disrupt viral capsids and render pathogens non-infectious. However, several critical knowledge gaps impede the widespread adoption of this technology. A more comprehensive understanding of the photoinactivation mechanisms of viruses, including their rapid mutagenicity and post-treatment viability, is essential for optimising photocatalytic disinfection protocols. While photocatalytic oxidation [1,2] offers an attractive solution for air purification by effectively decomposing various air pollutants into non-toxic byproducts, translating this technology into commercially viable photocatalytic reactors presents significant engineering challenges. The development of a robust, scalable, and efficient method for immobilising the photocatalyst onto a suitable support material remains the primary obstacle. Achieving successful and durable immobilisation under ambient conditions would represent a significant advance, accelerating the commercialisation and deployment of photocatalytic disinfection technologies for both surface and air decontamination. Although photocatalytic air purification offers numerous advantages, its practical application is often hampered by limitations, such as a relatively slow purification rate. Combining photocatalysis with complementary technologies, such as adsorption, photothermal catalysis (PTC), or plasma catalysis, presents a promising avenue for synergistic enhancement. Such hybrid approaches can improve overall treatment performance by, for example, facilitating the rapid capture of target compounds on the catalyst surface. PTC, in particular, combines the high efficiency and durability of thermal catalytic oxidation with the benefit of a lower energy consumption. Similarly, integration of plasma treatment can promote the degradation of air pollutants, while photocatalysis minimises the formation of potentially harmful byproducts. Despite these potential benefits, hybrid processes remain in their early stages of development [3,4], which requires further in-depth research to fully elucidate the underlying synergistic mechanisms and address the practical engineering challenges associated with their implementation. Hybrid technologies offer a promising approach for integration within heating, ventilation, and air conditioning (HVAC) systems to significantly mitigate the spread of viral infections, pathogenic microflora, and molecular air pollutants [5,6,7].

**Figure 1:**
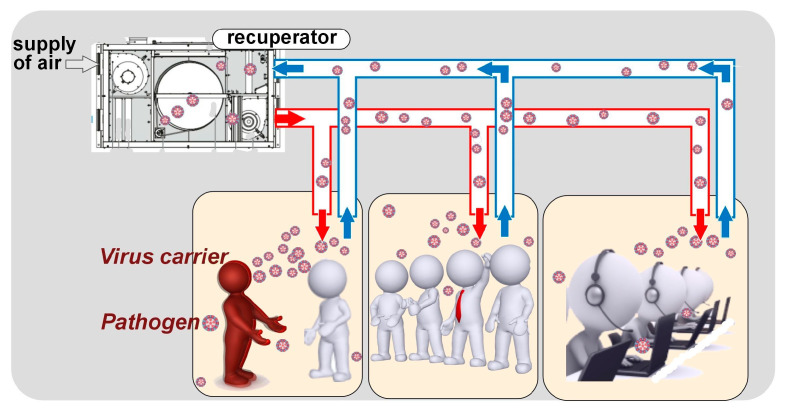
Pathways to the spread of pathogens in areas with centralised (HVAC) systems.

Public and administrative buildings commonly use supply and exhaust HVAC systems, often incorporating mechanical ventilation with coarse filters and rotary heat recovery devices. Although the main function of these HVAC systems is to maintain the indoor microclimate parameters within defined specifications [8,9,10], under certain operating conditions, they can inadvertently act as reservoirs and vectors for the accumulation and dissemination of airborne pollutants [11,12] (Figure 1).

Maintaining safe and healthy indoor air quality (IAQ) in buildings presents a complex challenge, as it is influenced by a multitude of interacting factors [13]. These include the intended use of the space, the architectural design and the specific characteristics of its ventilation system, the operating protocols used within the building, and the prevailing climatic conditions. Consequently, effective mitigation strategies require careful selection and appropriate integration of advanced air purification and disinfection technologies within heating, ventilation, and air conditioning systems (HVAC) [14].

**Figure 2:**
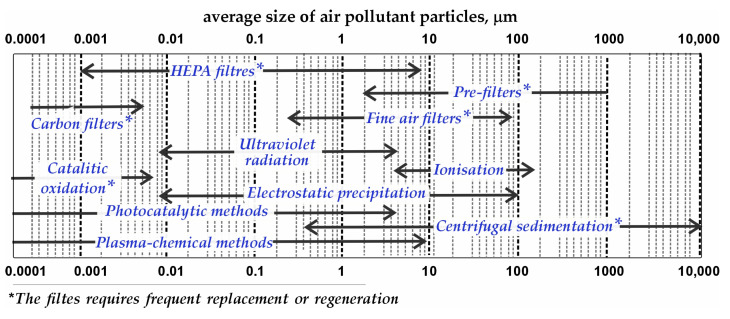
Operating ranges of current air cleaning and disinfection techniques.

The primary source of airborne pathogens within buildings is typically infected occupants whose respiratory activities generate infectious aerosols [15]. Pathogens can be introduced via outdoor air intakes, especially in regions with poor air quality. HVAC systems, although often employing recirculation for energy efficiency, can inadvertently contribute to the redistribution of these pathogens [16]. If recirculated air contains viable infectious agents, they can be drawn into the return air ductwork and subsequently disseminated throughout the building through the supply air stream. Although filters are standard HVAC components, their efficacy against all pathogens, particularly smaller viral particles, is limited [17]. Furthermore, the effectiveness of various available air purification methods (Figure 2) within HVAC systems can be substantially reduced by the limited residence time of aerosolised particles within the active zone under typical air exchange conditions [18,19,20,21,22,23,24,25,26].

A critical aspect of the performance of the HVAC system is filtration. Suboptimal filter selection, such as using filters with an inadequate Minimum Efficiency Reporting Value (MERV) rating for target pathogens, can significantly compromise the filtration barrier. Similarly, improper maintenance, including infrequent filter replacement or incorrect installation, can allow pathogens to penetrate the filter and enter the recirculated airstream. Furthermore, filters themselves can become reservoirs for microbial growth if not properly maintained, potentially exacerbating the problem. Beyond filtration, airflow dynamics and system hygiene play a crucial role in pathogen distribution. Poorly balanced airflow, often a consequence of suboptimal ductwork design or diffuser placement, can create stagnant zones that facilitate pathogen accumulation or, conversely, promote their widespread dissemination. Insufficient ventilation, characterised by an inadequate supply of fresh air, further compounds this risk by allowing pathogens to accumulate at higher concentrations. Inadequate system hygiene, including contaminated conduits and other components, such as cooling coils and drain pans, promotes microbial proliferation and can contribute to the overall burden of airborne pathogens.

Photocatalytic and plasmochemical air disinfection methods, frequently coupled with filtration systems for the removal and mitigation of ozone, have gained significant traction in contemporary applications due to their potential for controlling broad-spectrum pollutants [18,19]. However, translating these technologies, particularly photoplasma catalytic methods, to the dynamic aerodisperse flows characteristic of centralised ventilation systems presents substantial challenges [22,27]. Plasmochemical disinfection methods are inherently governed by the specific mechanisms and kinetics of the underlying plasmochemical reactions, which are further influenced by the unique chemical processes occurring within low-temperature plasmas and plasma jets as aerodisperse flows pass through them [21]. A more comprehensive understanding of these complex interactions is crucial to optimising the performance and reliability of these technologies in real-world HVAC applications.

The low-temperature plasma, generated via high-voltage discharge, interacts with these aerodisperse flows, and the discharge parameters directly influence the interaction time. Consequently, the design of the plasma-chemical treatment unit plays a critical role, directly affecting the intensity and duration of the plasma interaction with the organic molecules present in the air stream. A common by-product of plasma-based air treatment is the generation of excess ozone, which requires its reduction to levels below established allowed limits (e.g., 0.1 mg/m³ for occupational exposure) [28,29]. The efficacy of ozone abatement depends on the specific destruction mechanism employed, whether photolytic, thermal, or catalytic, each exhibiting different influencing factors [30,31]. For instance, the efficiency of catalytic ozone decomposition is modulated by the composition of the ozone–gas mixture, the design and contact surface area of the adsorber, the intensity of radiation (if applicable), temperature, humidity, airflow velocity, and the surface properties of the catalyst. Integrating photocatalytic filters within air purification systems offers several compelling advantages, including energy efficiency (low specific energy consumption), environmental benignity (decomposition into harmless by-products), and simplified maintenance requirements.

Finally, the efficacy of photocatalytic air purification is critically dependent on the confluence of factors related to the design and operation of the photocatalytic unit. The selection and strategic placement of the source of ultraviolet (UV) radiation is paramount, as the intensity and spectral characteristics of UV light directly influence the activation of the photocatalyst and the subsequent generation of reactive species responsible for pollutant degradation [18,28]. Furthermore, the aerodynamic resistance of the unit plays a significant role, affecting the airflow patterns and the residence time of pollutants within the reactor, thus affecting the overall purification efficiency. The material properties of the catalyst support matrix, including its composition and surface modification techniques used for photocatalyst immobilisation, are also crucial considerations [32,33,34,35,36]. These parameters influence pollutant accessibility, the rate of adsorption, and the efficiency of the photocatalytic reaction. A comprehensive understanding of the coupled heat and mass transfer phenomena within the porous catalyst structure is essential to optimise reactor performance [37,38]. Although energy conservation within HVAC systems has traditionally been approached through recirculating air exchange schemes incorporating rotary heat recovery [38,39], recent public health crises have led to a re-evaluation of these practices. Recommendations to limit the operation of the air conditioning system and minimise or eliminate indoor air recirculation have been implemented in some regions due to concerns about the transmission of airborne diseases. The absence of a harmonised international standard for HVAC systems that explicitly incorporates antipandemic measures underscores the need for further research and collaborative efforts to balance energy efficiency, indoor air quality, and public health considerations.

This article addresses the pressing need for effective air purification and disinfection strategies within building ventilation and air conditioning (HVAC) systems, particularly in light of increasing concerns about airborne transmission of infectious diseases. The prevalence of centralised ventilation systems in modern buildings, while offering advantages in terms of climate control and energy efficiency, can also inadvertently contribute to the rapid dissemination of airborne pathogens. Current approaches often do not provide adequate protection against these risks, highlighting a critical gap in existing HVAC technologies. Therefore, this work aims to develop and evaluate novel air purification and disinfection modules that integrate plasma chemistry and photocatalysis for seamless integration into centralised HVAC systems without requiring extensive system reconstruction. The overarching goal is to achieve a significant reduction in the pathogenic microflora, thus minimising the risk of infection and contributing to a healthier indoor environment. This research is particularly relevant given recent global health crises, which have underscored the vulnerability of indoor environments to airborne transmission and the urgent need for improved air disinfection strategies.

## 2. Materials and Methods

This section provides a detailed account of the experimental setup, materials, and procedures used to evaluate the performance of a pilot experimental air purification system. The primary objective of this study was to evaluate the efficacy of an integrated system, which combines plasma chemistry and filtration, in mitigating airborne particulate matter, organic compounds, and biological contaminant concentrations under controlled conditions, simulating real-world HVAC applications.

### 2.1. Pilot-Experimental Installation

The system was constructed using a modular architectural approach. This design principle facilitates ease of reconfiguration, allowing the integration of supplementary air purification technologies as needed. The modularity also allows for targeted maintenance and replacement of individual components, minimising system downtime, and maximising operational efficiency.

The functional modules were strategically placed at the intersection points of the air ducts. This placement was deliberate, with the aim of replicating the airflow dynamics and ensuring optimal contact between the air flow and the purification modules. This configuration ensures that air passing through the ventilation system is forced to pass through the purification modules, thus, maximising the efficiency of the air-cleaning process. The installation was equipped with a comprehensive suite of sensors designed to monitor critical operational parameters.

The instrumentation included a thermo-anemometer (CEM DT-3893, Shenzhen Everbest Machinery Industry Co., Ltd., located in Shenzhen, China) for real-time airflow rate measurement, providing volumetric flow data; a differential pressure transducer (MMC-200, Institute of Engineering Thermophysics of National Academy of Sciences of Ukraine, Kyiv, Ukraine) to assess aerodynamic resistance across modules; an air quality analyser (BauTech, Shenzhen, China) for temperature and relative humidity control; and a semiconductor ozone sensor (MQ-131, Zhengzhou Winsen Electronics Technology Co., Ltd., Zhengzhou, China) for environmental condition documentation.

**Figure 3:**
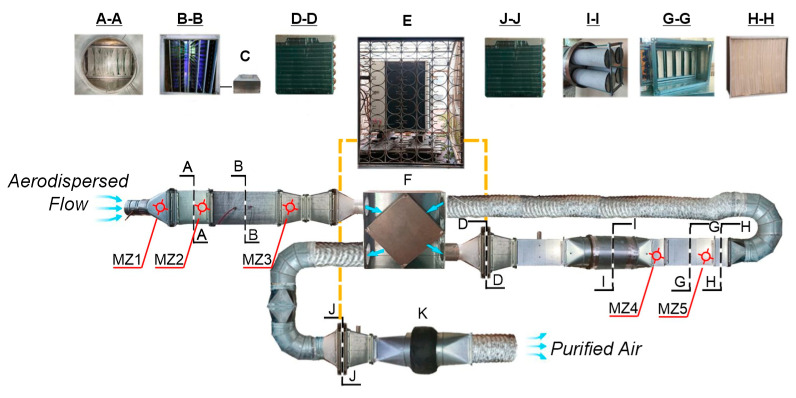
Schematic diagram and modular components of the experimental installation to investigate complex air purification and disinfection processes in ventilation systems: A—coarse filter; B—plasma ioniser; C—high voltage power supply; D—heat pump condenser; E—heat pump; F—recuperative heat exchanger; G—photocatalytic module; H—output filter; I—adsorption-catalytic module; J—heat pump evaporator; K—exhaust fan with performance control; MZ1–MZ5—air microflora monitoring zones.

Structurally, the installation comprised autonomous functional modules (Figure 3) strategically positioned within the air duct cross section to ensure representative airflow dynamics.

The pilot experimental setup allowed the variable sequencing of these modules within the air duct system. Each module was designed to target specific pollutant classes, employing different operational principles. The system incorporated a coarse filter (A) to remove mechanical particulates (dispersed particles > 0.4 µm), such as dust, soot, pollen, and animal dander, which can carry airborne biological pathogens. A cold plasma generator (B) induced physicochemical processes through low-temperature corona discharge, producing active oxygen (ozone). The plasma generation module included a high-voltage source (C) with adjustable output. Plasma treatment was designed to generate ozone concentrations that exceeded the allowed limits. Subsequently, the ozone-laden airflow, containing particles charged in the corona discharge zone, was heated in a plate cross-flow heat exchanger (F) and directed to an adsorption-catalytic module (I) for ozone decomposition. After the catalytic treatment, the air passed through an exhaust filter (G) before entering the ventilation system and ultimately the room. Temperature control within the air ducts was achieved using a heat pump-assisted circulation system (D↔E↔J). The airflow through the purification and disinfection system was regulated by a Vents VKM 315 fan (K) with a speed controller, providing a maximum air capacity of 1400 m^3^/h.

### 2.2. Construction of the Plasma-Chemical Air Treatment Module

**Figure 4:**
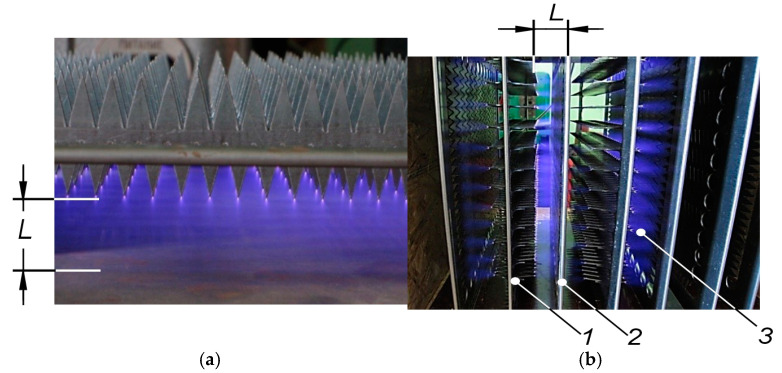
Design of the low-temperature plasma generation module: (**a**) stable low-temperature plasma discharge in a single section; (**b**) sectional arrangement of corona electrodes and counterpolarity plates within the low-temperature plasma generation unit: *1*—saw-toothed corona electrodes, *2*—plate electrodes, *3*—corona discharge glow; *L*—interelectrode distance (arc gap width).

The development of the plasma-chemical air purification module focused on the design and optimisation of a low-temperature plasma generator for efficient aerodisperse treatment. The core component of the module was a low-temperature plasma generator (Figure 4), connected to a high voltage power supply with an adjustable output voltage.

The structural elements of the corona discharge generation section (Figure 4a) within the plasma-chemical module were mounted in a housing, forming a cassette assembly (Figure 4b). A plasma-chemical module was developed with the following technical parameters and operating conditions: overall dimensions: 320 × 260 × 260 mm; interelectrode distance: 20–25 mm; voltage applied to the low-temperature plasma unit: up to 20 kV; electric field intensity between electrodes: up to 8 × 10^3^ V/cm; air exchange rate: up to 1400 m^3^/h; operating temperature range: 17–30 °C; relative humidity of the air: 45–70% at 25 °C.

**Figure 5:**
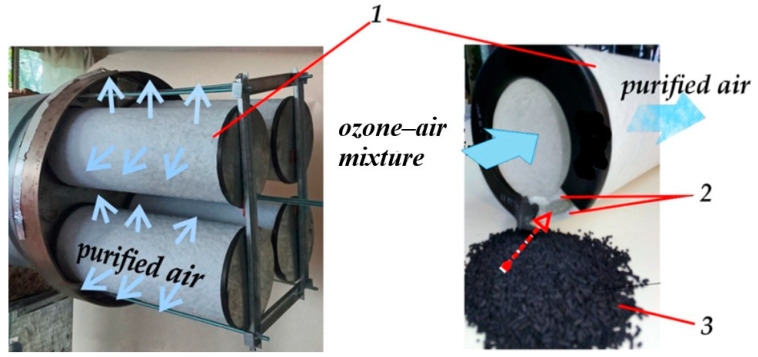
Adsorption-catalytic module: *1*—cartridge, *2*—filter layers, *3*—adsorption-catalytic granular layer.

Following plasmachemical purification, the air stream enters an adsorption-catalytic module (Figure 5), which consists of a set of cartridges housed within a box. Each cartridge is composed of two layers of G-class filter material, and the space between them is filled with adsorbent granules.

The granules are formed from a mixture of carbon particles and calcium aluminate. Aerodisperse particles become electrically charged within the corona discharge zone and subsequently deposit and are electrostatically retained on the surface of filter layer *2* (Figure 5). Thus, the filter surface acts as a barrier, preventing solid particles from reaching the surface of adsorbent-catalyst *3*. The porous surface of the adsorbent adsorbs molecular compounds and excess ozone molecules, followed by degradation via heterogeneous catalysis.

### 2.3. Construction of Tablet-Type Photocatalytic (PC) Air Purification Modules

**Figure 6:**
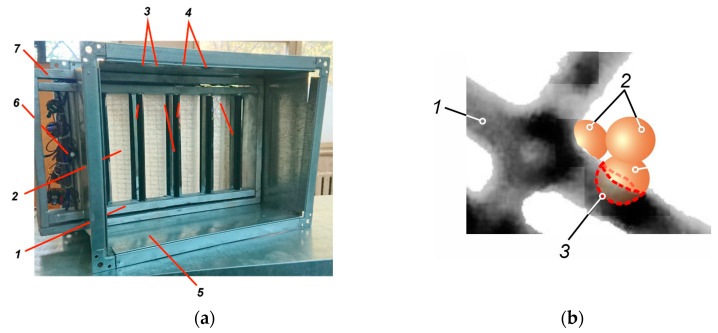
Photocatalytic air disinfection module: (**a**) *1*—PC cassette; *2*—PC filter; *3*—UV generator; *4*—reflective screen; *5*—filter box; *6*—UV lamp control unit; *7*—mounting frame. (**b**) Components of the PC filter: *1*—polypropylene matrix fibre support; *2*—photocatalytic particles; *3*—interface between fibre matrix/embedded particles.

Tablet-type PC air purification modules (Figure 6) were constructed to facilitate photocatalytic air treatment within ventilation systems.

Each module consisted of a PC filter and UV emitters, assembled within a Z-line frame. To allow easy maintenance and replacement, the modules were designed for installation within standard filter boxes in the ventilation system.

The UV-radiation generators (*3*) were strategically positioned to ensure maximum direct illumination of the photocatalytic surface, maintaining an optimal radiation intensity of 1.2–2.0 mW/cm^2^.

Furthermore, a polished aluminium screen (*4*) was incorporated into the module design to enhance illumination. This screen, which reflects up to 80% of light within the 300–400 nm wavelength range, provided additional illumination through reflected light, thus maximising the efficiency of the photocatalytic process.

### 2.4. Structural and Topological Characterisation of the AEROXIDE^®^ TiO_2_ P25 Catalyst Layer

The structural and topological characteristics of the AEROXIDE^®^ TiO_2_ P25 (Evonik Operations GmbH, Essen, Germany) catalyst layer deposited on the carrier fibres of the polymer matrix were evaluated using scanning electron microscopy (SEM). The SEM analysis was performed at an accelerating voltage of 10 kV to visualise and assess the morphology and distribution of the catalyst on the individual fibres of the support matrix.

### 2.5. Scanning Electron Microscopy (SEM) Analysis of Adsorbent Granules

To comprehensively characterise the adsorbent granules, scanning electron microscopy (SEM) was employed. This technique was used to assess several key morphological and compositional attributes, including granule surface morphology, uniformity of component distribution, and mixture dispersity. These analyses were performed using a JEOL Auger Micro Probe JAMP 9500F (JEOL Ltd., Akishima, Tokyo, Japan). This instrument provides high-resolution imaging and elemental analysis, enabling a detailed characterisation of the adsorbent granules.

### 2.6. Surface Area Analysis

The specific surface areas (S_BET_) of the samples were evaluated using the standard Brunauer–Emmett–Teller (BET) method for nitrogen adsorption data. To ensure accurate measurements, the synthesised adsorption catalyst pellets were degassed overnight at 250 °C under vacuum. Subsequently, nitrogen adsorption–desorption measurements were performed at 77 K, covering a relative pressure range (p/p_0_) from 0.01 to 0.99, using a Micromeritics ASAP 2020 bulk adsorption analyser (Micromeritics Instrument Corporation, Norcross, GA, USA).

### 2.7. Methodology to Assess Microbiological Air Pollution

The evaluation of microbiological air pollution was carried out using both impaction and sedimentation methods to collect airborne microorganisms in culture medium [40,41]. Meat peptone agar (MPA), a standard medium suitable for the cultivation of a wide range of microorganisms, was selected as the culture medium. During the experimental studies, air was drawn through the pilot plant at a controlled flow rate. Sterile Petri dishes containing MPA were placed at designated monitoring points (Figure 3).

A qualitative assessment of the inhibition of fungal growth of eukaryotic organisms (mould fungi) was performed at each stage of the aerodisperse medium disinfection process. Selected air microflora samples collected from the monitoring points were incubated on a thermostat at 37 °C for 142 h.

## 3. Results

### 3.1. Functional Characteristics and Performance of the Pilot Experimental Air Purification System

A pilot experimental installation was specifically designed and constructed to mimic the operational conditions of a centralised HVAC system. This installation was characterised by its modular design, incorporating autonomous functional units strategically positioned at the intersection of air ducts to replicate representative airflow dynamics. This design allowed for sequential air treatment, with each module targeting specific classes of pollutants.

Using a modular design, the centralised HVAC system was engineered to allow flexible configurations and seamless integration of additional air purification technologies. The placement of functional modules at air duct intersections was meticulously planned to replicate representative airflow dynamics and maximise contact between the air stream and purification elements. To allow precise control and data acquisition, the installation was equipped with a suite of sensors that continuously monitor airflow rate, pressure drop, temperature, and humidity.

The products of high-voltage electric discharge, including ozone (O_3_), atomic oxygen (O), excited molecular oxygen (O_2_), hydroxyl radicals (OH), and ions, exhibit a high oxidative capacity. The generated hydroxyl radicals and ozone react with organic molecules through hydrogen extraction, forming alkyl radicals that are subsequently rapidly oxidised in the air stream. This mechanism facilitates the degradation of a wide range of organic compounds, including those that make up living organisms. Critically, it effectively disrupts the cell walls and bacterial capsules of pathogenic microorganisms. Furthermore, the bombardment of the membranes of organic molecules with electrons upon contact with the ionised plasma volume contributes to their structural breakdown.

Within the photocatalytic (PC) module, the air purification process proceeds through the following stages [42]:

− Adsorption of microorganisms and harmful substance molecules onto the PC surface, coupled with the generation of oxidising agents on the photocatalyst surface under UV irradiation (365–405 nm);
− Degradation of the entire molecular structure of microorganisms via interaction of their organic matter with photoinduced radicals on the catalyst surface, resulting in complete inactivation and deactivation of gas-phase compounds and aerosols at the molecular level;
− Complete conversion of microbial or other hazardous pollutant substances into elementary inorganic compounds, yielding harmless byproducts.

To intensify the process of decomposition of excess ozone, the air is heated before being fed into the catalytic filter, which helps to increase the efficiency of the ozone destruction process on the catalyst surface at temperatures above 50 °C [31]. Furthermore, maintaining an elevated air temperature within the device promotes the decomposition of organic impurities that accumulate on the surface of the catalytic filter [43].

**Figure 7:**
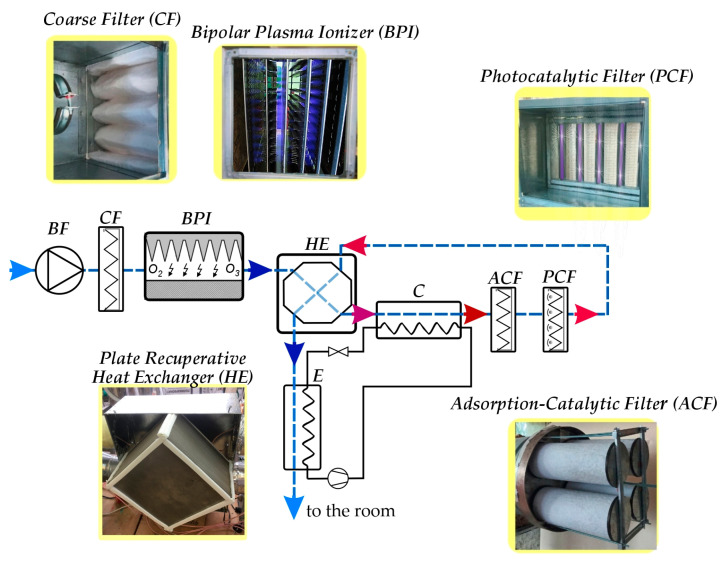
Block diagram of energy-efficient air purification and disinfection: *BF*—blower fan; *CF*—coarse filter; *BPI*—bipolar plasma ioniser; *HE*—heat exchanger (recuperator); *ACF*—adsorption-catalytic module; *PCF*—photocatalytic module; *C*—heat pump condenser; *E*—heat pump evaporator.

The technological scheme of the air purification and disinfection process with the combined catalytic–thermal decomposition system of excess ozone is shown in Figure 7.

**Figure 8:**
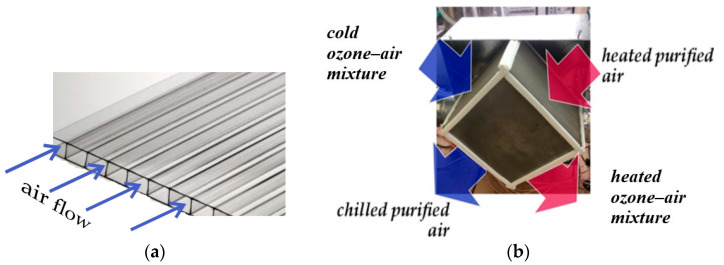
Polymer cross-fin plate (**a**) and recuperative heat exchanger for air purification system (**b**).

In centralised ventilation systems utilising rotary heat recovery units, there is a potential for partial air transfer from the exhaust pipe to the supply duct. This phenomenon can facilitate the dissemination of pathogenic microflora throughout the building. Plate-type recuperators, which achieve heat transfer through a solid heat exchange surface, eliminating airflow mixing, address these limitations. For the air purification system developed in this study, a polymer cross-flow plate recuperator was designed and subsequently tested (Figure 8).

The heat exchange surface of the recuperator was constructed from finned plates with a wall thickness of 0.15 mm and a fin height of 2 mm. These plates were assembled in a heat exchange package, forming cross channels with dimensions of 6 × 2 mm. The performance of the recuperative heat exchanger was evaluated through a series of controlled laboratory tests. The results demonstrated that at an air velocity of 0.75 m/s within the channels, the recuperator achieved a thermal efficiency of 0.9. However, as the air velocity increased to 3 m/s, the thermal efficiency decreased to 0.6. These tests were carried out under controlled conditions, simulating typical operating parameters of ventilation systems.

The energy consumption for thermal air treatment was significantly reduced through heat recovery, utilising a heat pump in conjunction with an air–air recuperative heat exchanger. This approach facilitated efficient temperature maintenance for both heating and cooling, achieving a 7–8-fold reduction in heating energy requirements for aerodispersion. With a recuperative heat exchanger efficiency of 0.65–0.70 and a heat pump coefficient of performance (COP) of 2.7–3.2, the integrated plasma-chemical and photocatalytic air purification system, coupled with a heat pump-driven ozone–air mixture heating to 70 °C, demonstrated an energy consumption of up to 2 Wh/m^3^.

Field tests of the developed equipment, which was installed in the existing ventilation system with a rotary recuperator and forced air injection, were carried out in the autumn–winter period in a production room measuring 24 × 12 m, with a height of 6 m. The air exchange rate was provided at level 2. The maximum number of workers who were in the production room at the same time was 12 people. The average temperature and relative humidity in the room were 14–16 °C and 65–70%, respectively.

The disinfection system was carried out for 10 days during working hours. During the operation of the plasma-chemical and photocatalytic air disinfection modules, air sampling for the culture medium was carried out at four points in the room at a height of 1.5 m from the floor. For a comparative analysis of the microflora content in the air, the samples with the largest contamination area after two days of exposure in the thermostat at 37 °C were selected.

### 3.2. Characterisation and Fabrication of Photocatalytic (PC) Modules

#### 3.2.1. Material Selection and Characterisation

For the photocatalytic material, highly dispersed crystalline titanium dioxide (TiO₂) AEROXIDE^®^ TiO_2_ P25 (Evonik Operations GmbH, Essen, Germany) was chosen. This material, characterised by nanoparticles ranging from 10 to 45 nm, exhibited a specific surface area of 50 ± 15 m^2^/g. The TiO_2_ powder consisted of a mixed phase composition, with 80–90% anatase and 10–20% rutile phases, as previously documented [35].

A nonwoven fabric composed of polypropylene (PP) fibres served as the support matrix for the photocatalyst. This fabric was selected due to its inherent macroporous structure, which provided an initial aerodynamic resistance of 10–30 Pa. This selection strategy was designed to minimise the increase in aerodynamic resistance that would occur during surface modification, as it was expected that micropores and partially mesopores would be occluded by the deposited catalyst layer [44,45].

#### 3.2.2. PC Module Fabrication Process

A multistage fabrication process was used to create PC modules on polypropylene membranes. This process was designed to optimise the performance characteristics of PC filters by precisely controlling the surface modification of the carrier matrix. The fabrication process included the following steps: formation of the matrix geometry and composition, surface modification of the carrier matrix by associated processes, integration of ultraviolet (UV) generators, and assembly of the photocatalytic module. The carrier matrix consisted of two layers of fibrous polypropylene (PP) membrane with a metal frame between them for structural rigidity. Due to corrugation with a corrugation opening angle of 90°, surface development was achieved 1.4 times. After matrix formation, the photocatalyst was applied on the surface. In this study, a modification method was applied that combined immersion in a 3% dispersive hydrogen medium with a catalyst at a fixed temperature, followed by filtration through the porous space of the matrix. The modification was carried out in an innovative unit of type PF-200. Its operation is based on the principles of discrete pulse energy injection into heterogeneous systems [45,46,47,48].

### 3.3. Effect of Electrode Geometry and Inter-Electrode Distance on Corona Discharge Characteristics

**Figure 9:**
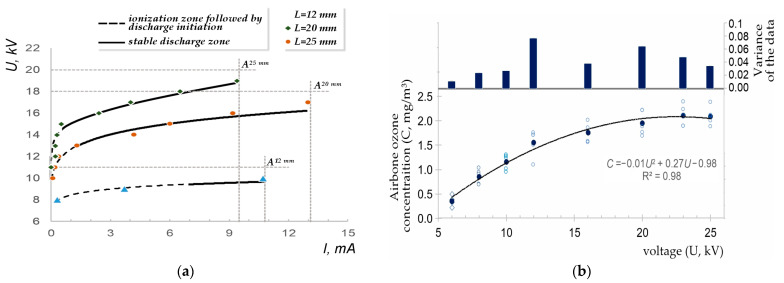
Experimental determination of stable operating parameters for low-temperature plasma generation: (**a**) generator voltage-current characteristics (VACs) at variable *L*; (**b**) ozone synthesis (post-generator at MZ3) as a function of applied voltage at an air velocity of 1.2 m/s and a temperature of 18–20 °C. *A*—air-gap breakdown points; *L*—interelectrode distance (arc gap width).

The determination of stable operating parameters for the low-temperature plasma generator involved evaluating various corona electrode geometries. Saw-toothed electrodes were found to optimise ionization and electric field intensity. Voltage-current characteristics (VACs) were measured at varying interelectrode distances to define these parameters (Figure 9a).

A systematic investigation into the optimisation of stable corona discharge was conducted, evaluating various geometries of corona electrodes. Optimal ionisation and electric field intensity within the plasma-chemical treatment zone were achieved utilising saw-toothed electrodes. The relationship between current and applied voltage across the corona electrodes was determined at varying interelectrode distances. Stable operating parameters for the low-temperature plasma generator were established through analysis of its voltage-current characteristics (VAC) (Figure 9b). The operational range of the high-voltage potential, which facilitates stable corona discharge and air-gap ionisation, was defined as a function of the interelectrode distance (*L*). Air-gap breakdown, indicated by arc ignition and ionisation cessation, was observed by incrementally increasing the applied voltage. The onset of air-gap breakdown was recorded as a function of interelectrode distance and applied voltage on the VAC curves (points *A*). Experimental results demonstrated a significant influence of interelectrode distance on the stability of the low-temperature plasma. Specifically, an interelectrode distance of 20–25 mm was found to broaden the stable corona discharge range across varying high-voltage levels supplied to the plasma generator.

### 3.4. Adsorbent-Catalyst Characteristics in the Air Stream Purification Adsorption-Catalytic Module

The resulting SEM micrographs were analysed to determine the average particle size, the pore size distribution, and the surface area of the adsorbent granules. Elemental analysis was performed using energy-dispersive X-ray spectroscopy (EDS) to confirm the composition and distribution of the components.

**Figure 10:**
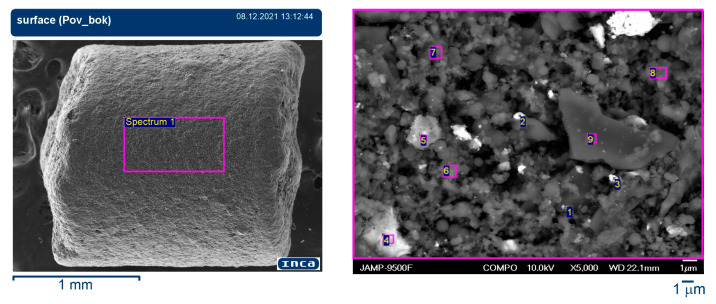
Scanning electron microscopy (SEM) images of the adsorbent-catalytic granule: overview at ×50 magnification (**left**) and detailed surface morphology spectrum 1 at ×5000 magnification (**right**).

The catalytic activity of the adsorbent is intrinsically linked to the development of its granular surface area. Analysis of the adsorption-catalytic granular layer (Figure 10) revealed a favourable distribution of aggregates formed from the mixture of components, resulting in a predominantly mesoporous structure, with an accessible internal surface. This structure facilitates efficient mass transfer of aerodispersed species and reaction products containing ozone.

The Brunauer–Emmett–Teller (BET) method determined a maximum specific surface area of 180–280 m^2^/g, enhancing the adsorption capacity and catalytic reaction rates. Furthermore, the microanalysis of the surface of the adsorption-catalytic granule, as depicted in the spectrum (Figure 10), revealed the elemental composition presented in Table 1.

The percentages of key elements varied at different measurement points, indicating the distribution of the components within the granule. In particular, carbon (C) was the predominant element, with varying levels of oxygen (O), aluminium (Al), silicon (Si), and calcium (Ca) also detected. Variability in elemental composition at different points suggests a heterogeneous distribution of components within the granule, which may influence its catalytic properties.

### 3.5. Characterisation of Deposited TiO_2_ Catalyst Layer and Aerodynamic Drag Analysis

The structural and topological characteristics of the AEROXIDE^®^ TiO_2_ P25 catalyst layer deposited on the fibres of the polymer matrix-carrier were evaluated using scanning electron microscopy (SEM) at an accelerated voltage of 10 kV.

**Figure 11:**
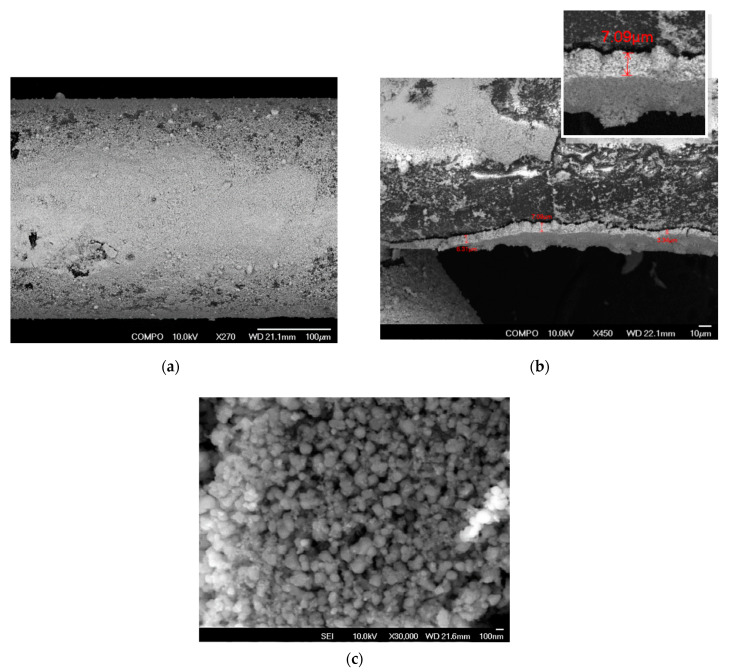
Cross-sectional SEM topography of the TiO_2_ matrix coating, illustrating surface features at (**a**) 270×, (**b**) 450×, and (**c**) 30,000× magnifications.

*Catalyst Layer Morphology.* The PP matrix modification technology resulted in a uniformly rough coating surface (Figure 11a,b). The deposited catalyst layer exhibited a thickness ranging primarily from 5 to 10 μm (Figure 11c). The texture was corpuscular, formed by aggregated titanium dioxide particles with sizes between 80 and 160 nm.

The analyses demonstrated that the monodisperse aggregates formed a predominantly monopore space. This specific surface texture maximised the accessibility of UV radiation to the titanium dioxide particles, thereby enhancing the photoreaction surface area.

**Figure 12:**
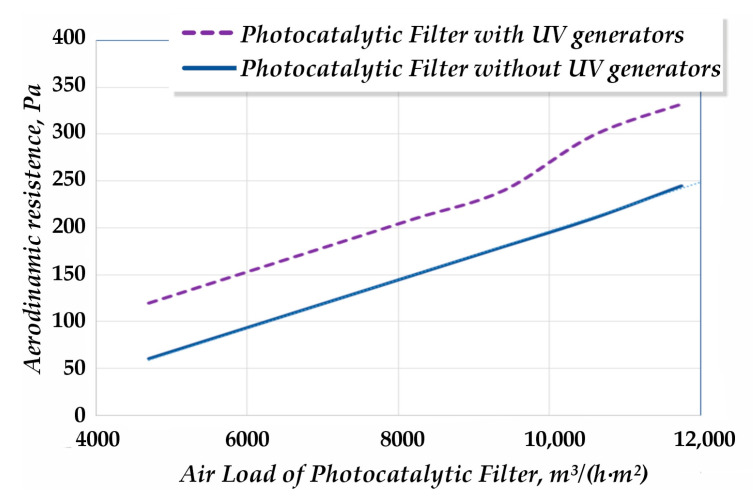
Aerodynamic characteristics of the photocatalytic air disinfection module.

*Aerodynamic Drag Analysis.* The proposed geometric parameters of the filter were designed to increase the contact surface area while minimising the aerodynamic drag of the PC module. A comparative analysis of the modified PC filter was performed, both with and without installed UV emitters. The results showed an increase in aerodynamic drag by a factor of 1.3 to 2, depending on the air load (Figure 12).

This increase was attributed to the presence of UV emitters and their associated support structures within the filter module.

### 3.6. Microbiological Air Pollution Analysis Results

During the experimental studies, air was drawn through the pilot plant at a controlled flow rate. Sterile Petri dishes containing meat peptone agar (MPA) were placed at designated monitoring points (Figure 3).

**Figure 13:**
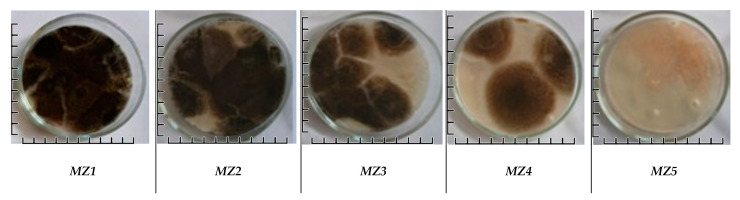
Visualisation of viable mould fungus colonies grown from samples taken at locations detailed in Figure 4. Each image includes a 10 mm scale bar.

A comparative analysis of the samples was performed, which included qualitative and quantitative visual evaluations (Figure 13).

The experimental data for the normal operating mode are shown in Table 2, with the following parameters:

− Average airflow velocity in the central air conduit—5 m/s;
− Average airflow temperature—17 ℃;
− Voltage supplied to the ozone generation unit—20 kV.

## 4. Discussion

**Figure 14:**
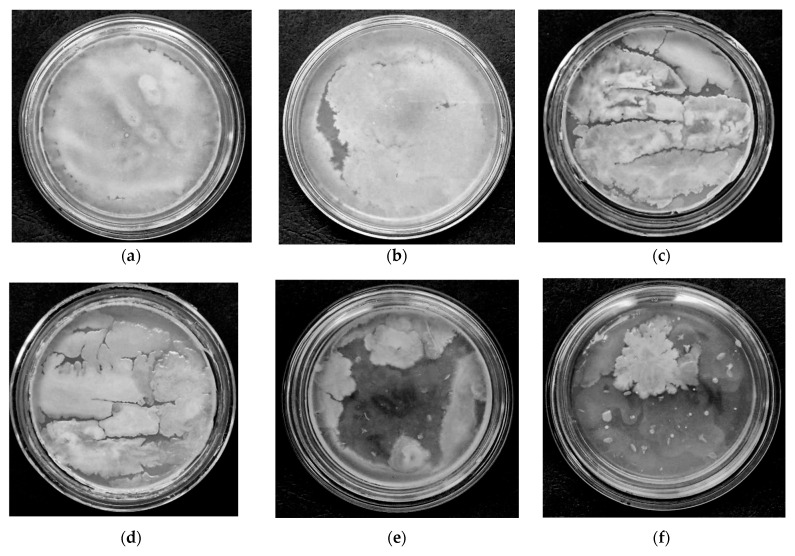
Samples of indoor air microflora: (**a**) initial (before starting the plant); (**b**) after 6 h of plant operation; (**c**) after 9 h; (**d**) after 10 h; (**e**) after 18 h; (**f**) after 28 h of plant module operation.

The results of microbiological studies of air quality (Figure 14) showed that a decrease in the number of microflora was already observed after the first day of equipment operation (Figure 14c). After the third day of plant operation, no significant changes were detected in the samples taken.

During the microflora testing period, the airborne microflora content decreased by 60% after 3 days of unit operation. In addition, the microflora content did not decrease, which may have been caused by the design flaws of the applied recuperator.

The results of laboratory studies and field tests showed that the depth and speed of aerodisperse microflora inactivation depend mainly on the volume concentration of atomic oxygen and ozone. The possibility of increasing the concentration of ozone in the plasma-chemical air treatment module is limited by the ability of the adsorption-catalytic unit to convert excess ozone into molecular oxygen before air supply to the room.

## 5. Conclusions

This study provides compelling demonstration of the efficacy of a synergistic plasmochemical and photocatalytic approach, complemented by a catalytic–thermal ozone decomposition system, in achieving substantial reductions in molecular pollutants and airborne microbial contamination to levels deemed safe. The contribution to mitigating infectious diseases is robustly supported by both controlled laboratory experiments and real-world field trials, which consistently demonstrated the operational stability of the proposed disinfection modules.

However, a critical analysis of the study results revealed key determinants influencing the overall efficiency of ozone-mediated disinfection. In particular, the concentration-time product emerged as the primary limiting factor in the disinfection of bioaerosol-laden airflows. Although the developed plasma-chemical generator achieved ozone concentrations exceeding 50 times the maximum permissible concentration (MPC), the transient nature of high-speed airflows limited the contact time between biopollutants and ozone to mere seconds, or even subsecond intervals.

Furthermore, the inherent safety constraints associated with residual ozone levels in the output air, as defined by the MPC, necessitate the optimisation of the adsorption-catalytic module. Current research initiatives are focused on the development and evaluation of novel composite granule formulations, optimised structural configurations, particle sizes, and improved mechanical integrity under high frontal airflow conditions. Similarly, the reliability of the photocatalytic module is being addressed through investigations into improving the durability of the TiO_2_ photocatalyst layer and exploring the feasibility of replacing traditional gas-discharge UV emitters with more energy-efficient LED alternatives. Future advances in plasma-photocatalytic air purification for dynamic airflow systems hinge on the precise harmonisation of technological and operational parameters, including airflow velocity, temperature, humidity, and module aerodynamic resistance. Achieving an optimal balance between purification efficacy and the overall energy efficiency remains a paramount objective. Despite these identified challenges, the implementation of UV-mediated air purification, which mimics natural oxidative processes, presents a promising avenue for advanced air treatment. Continued research on mitigating practical limitations, such as photocatalyst fouling/deactivation, simplifying catalyst immobilisation, and developing efficient reactor designs, is essential to translate laboratory findings into scalable, real-world applications. The results of this study offer a viable route for retrofitting existing building ventilation and air conditioning systems with advanced modules capable of inactivating and removing molecular pollutants. This technology addresses a significant and growing need in both temporary (e.g., administrative buildings, retail establishments, clinics) and permanent occupancy settings (e.g., corporate offices, educational institutions, healthcare facilities), thereby creating substantial opportunities for the integration of the proposed equipment into centralised HVAC systems.

## Figures and Tables

**Table 1:** Elemental composition of the adsorption-catalytic granule spectrum 1 (weight %).

Point	C	O	Na	Mg	Al	Si	S	K	Ca	Fe
1	74.60	17.21	0.00	0.02	2.49	3.16	0.23	0.00	2.29	0.00
2	55.12	13.67	0.06	1.82	2.27	10.20	0.04	0.00	09.02	7.82
3	66.38	21.32	0.09	0.22	3.54	3.93	0.20	0.14	02.03	2.14
4	39.80	33.75	0.99	0.43	11.01	10.74	0.06	0.50	1.16	1.56
5	78.80	12.59	0.00	0.95	0.54	1.10	2.63	0.00	0.71	2.68
6	94.63	3.78	0.00	0.03	0.39	0.47	0.38	0.02	0.24	0.07
7	97.29	1.99	0.08	0.00	0.14	0.08	0.27	0.00	0.16	0.00
8	95.53	2.86	0.03	0.08	0.39	0.56	0.45	0.03	0.06	0.00
9	96.58	2.84	0.00	0.01	0.00	0.07	0.42	0.07	0.00	0.00

**Table 2:** Characteristics of the MZ aerodisperse system at the monitoring point.

Parameters	Monitoring Points				
duration of the purification process: 30 min	MZ1	MZ2	MZ3	MZ4	MZ3
content of dispersed particles smaller than 2.5 µm, µg/m^3^	30.0	15.0	13.0	9.0	8.0
ozone concentration, mg/m^3^	0.000	0.153	4.6	0.342	0.095
relative humidity, %	58.0	58.0	58.0	57.0	56.0

## Data Availability

The original contributions presented in this study are included in the article. Further inquiries can be directed to the corresponding author.
